# Analysis of different genotyping and selection strategies in laying hen breeding programs

**DOI:** 10.1186/s12711-025-00948-4

**Published:** 2025-04-07

**Authors:** Lisa Büttgen, Henner Simianer, Torsten Pook

**Affiliations:** 1 Animal Breeding and Genetics Group, Department of Animal Sciences, Center for Integrated Breeding Research, University of Goettingen, Albrecht-Thaer-Weg 3, 37075 Goettingen, Germany; 2 Animal Breeding and Genomics, Wageningen University & Research, P.O. Box 338, 6700 AH Wageningen, The Netherlands

## Abstract

**Background:**

Genomic selection has become an integral component of modern animal breeding programs, having the potential to improve the efficiency of layer breeding programs both by obtaining higher prediction accuracies and reducing the generation interval, particularly for males, who cannot be phenotyped for sex-limited traits such as laying performance. In the current study, we investigate different strategies to reduce the generation interval either for both sexes or only for the male side of the breeding scheme based on stochastic simulation using the software MoBPS. Additionally, prediction accuracies based on varying proportions of genotyping and phenotype- and pedigree-based selection as well as genomic breeding values are compared.

**Results:**

Selection of hens based on estimated breeding values, either pedigree-based or genomic, increased genetic gain compared to selection based on phenotypes only. The use of two time-shifted subpopulations with exchange of males between subpopulations to reduce the generation interval on the male side led to significantly higher genetic gains. Reducing the generation interval for both males and females was only efficient when population sizes were maintained, which result in doubling of the number of females to genotype and phenotype within the same time frame compared to the scenarios with the longer generation intervals. Although substantially higher gains were obtained by in particular pedigree-based selection of females and a reduction of generation intervals this led to substantially greater rates of inbreeding per year. The use of a genomic relationship matrix in breeding value estimation instead of a pedigree-based relationship matrix not only increased genetic gains but also reduced inbreeding rates. The use of optimum contribution selection led to basically the same genetic gains as without it but reduced inbreeding rates. However, overall differences obtained with optimal contribution selection were small compared to differences caused by the other effects that were considered.

**Conclusions:**

The reduction of the generation interval on the male side by the use of genomic estimated breeding values was highly beneficial. Reduction of the generation interval on the female side was only beneficial when a high proportion of hens was genotyped and housing capacities were increased. On the female side of a layer breeding program, selection based on pedigree-based estimated breeding values was inferior to phenotypic selection, as it resulted in a substantial increase in inbreeding rates.

**Supplementary Information:**

The online version contains supplementary material available at 10.1186/s12711-025-00948-4.

## Background

Breeding programs for farm animals have continuously improved over the last decades and genomic selection has become a widely used application for practical breeding, also in poultry [1]. In other livestock species, particularly dairy cattle, genomic selection drastically reduced the generation interval and additionally saved the costs of progeny testing [2] by the use of genomic instead of pedigree-based estimated breeding values using best linear unbiased prediction (BLUP) [3].

Approaches to optimize animal breeding programs often focus on breeding value estimation and selection of males, as higher selection intensities are commonly applied on the male side. Compared to the use of genomic selection of males, the potential of genotyping and genomic selection of hens has not been studied extensively. Wolc et al. [4] have shown exemplarily that for genomic selection in layers, single-step genomic BLUP (ssGBLUP) [5, 6] is the best choice in a setting with genotyped and non-genotyped individuals, due to the higher accuracies achieved compared to estimated breeding values (EBVs) based on pedigree-based BLUP.

Genotyping and genomic selection allow for shortening generation intervals, as reliable breeding values can be estimated for young animals that are not yet phenotyped at the time of selection. As the relative cost of genotyping in relation to housing and phenotyping is much higher in layers compared to dairy cattle, the economic potential of reducing the generation interval in layers is considered lower [1, 2, 7]. Nonetheless, shortening generation intervals could still lead to a higher genetic gain per time unit [8], but so far, this potential was not quantitatively evaluated. Such an analysis is further complicated by the need to account for various aspects such as its impact on genetic diversity or on the accuracy estimated breeding value, as animals are selected before own phenotypes or phenotypes of siblings or offspring are available. While typical laying hen breeding programs as described by Sitzenstock et al. [9] have a generation interval of about 14.5 months, it is biologically possible to shorten the generation interval by more than half. The use of genomic selection allows selection at begin of the reproductive age which results in generation intervals of 6 months [10, 11].

As selection of females at the beginning of their reproductive age would mean that females are selected before own phenotypes are generated and because within family selection is difficult without own phenotypes or genomic information, Wolc et al. [12] suggested a breeding scheme for layers that reduces the generation interval only on the male side and the use of genomic selection. Not shortening the generation interval for females enables the use of phenotypes of the female selection candidates in order to improve their selection accuracy. In terms of reproductive traits, this also avoids selection pressure on early sexual maturity of hens and sub-optimal egg sizes [12]. This approach might, however, lead to higher rates of inbreeding per year than with equal generation intervals for males and females, since having different generation intervals for males and females requires the breeding program to be restructured, but this was not considered in the study of Wolc et al. [12].

Management of genetic diversity and controlling inbreeding are important components of breeding program design to avoid adverse long term effects on the breeding program. On the one hand, a reduction of genetic diversity leads to a loss of the potential for adaptation of populations to currently unknown future conditions [13]. On the other hand, inbreeding has been reported to affect reproduction-related traits [14, 15] and important production traits [14, 16] in layers. As shown by König et al. [17], applying optimum genetic contribution selection [18] to layer breeding programs either allows the rate of inbreeding to be reduced while maintaining the rate of genetic gain or to obtain higher genetic gains while constraining the inbreeding rate.

Quantitative analysis by deterministic calculations can aid in the analysis of breeding program design and provide a basis for future decisions. Sitzenstock et al. [9] previously analyzed the potential of genomic selection in laying hen breeding by modelling various scenarios in the breeding plan software ZPLAN+ [19]. ZPLAN+ uses a deterministic approach to calculate the expected outcomes of a breeding program such as the expected genetic gain and inbreeding rates, based on the gene flow model [20, 21] and quantitative genetics theories [8]. Based on these models, Sitzenstock et al. [9] showed that genomic selection can be used in an established layer breeding program either to reduce the generation interval or to increase the accuracy of the selection. However, one of the main limitations of these deterministic calculations is that the accuracy of the genomic EBVs can only be approximated using the size of the calibration set [22], without taking population structure, age structure and phenotyping structure into account. Consequently, it is extremely challenging to analyze complex breeding programs with overlapping generations using deterministic methods, e.g. when shortening the generation interval only for males, which are not being phenotyped for most traits in laying hen breeding programs. The outcomes of such deterministic models depend highly on the assumptions made. E.g., expected genetic gain from the breeder’s equation [8] increases linearly with the assumed accuracy of the EBVs. Also with regard to inbreeding, deterministic models quickly become highly complex for realistic breeding programs and struggle to generalize [23] or are limited in modelling the impact of selection with multiple generations [20, 24].

In contrast, stochastic simulation, using tools such as MoBPS [25] or AlphaSimR [26], provides a more flexible approach to model breeding programs and account for population structure as well as age structure, and phenotyping structure of the population in breeding value estimation. As individual genotypes and phenotypes are simulated, the accuracy of EBVs is not derived using deterministic formulae [22, 27] but can be empirically extracted from the correlation of true and estimated breeding values. This accounts directly for complex population structures with overlapping generations and allows the true breeding value and inbreeding level to be known for each individual [25].

In the current study, different scenarios of a typical laying hen breeding program were simulated stochastically to assess the impact of the use of genomic EBVs for females and of shortening generation intervals. For this, shortening the generation interval by the selection of animals before phenotypes on female selection candidates are available [2] was considered, either for both males and females or for males only. Furthermore, the effect of genotyping females was evaluated. The different scenarios were compared in terms of genetic gains, development of inbreeding, and accuracies of EBVs.

## Methods

### Simulations

The simulations were based on the general laying hen breeding program used by Sitzenstock et al. [9] as a reference, also adopting their values for heritabilities, phenotypic standard deviations (SD), correlations between the traits, and economic weights (Table 1). For simulation of the breeding programs, the software MoBPS [25] was used, with all simulation scripts entered using the associated web-based interface [28] [see Additional file 1 Input files S1 to S18]. The initial breeding population of 5500 females and 800 males was generated by simulating 10 generations of random mating between a pool of 1000 females and 200 males. A total of 50,000 single nucleotide polymorphisms (SNPs) were simulated for each animal based on a randomly sampled subset of the Affymetrix Chicken600K Array [29]. In total nine traits were simulated: laying performance, divided into four time periods, egg weight, feed consumption, egg shell strength, hatchability, and mortality [9]. All traits were simulated as polygenic traits based on 1000 additive quantitative trait loci (QTLs) with effect sizes sampled from a gaussian distribution with mean 0 and same variance for each QTL. The QTLs were sampled from the simulated SNPs and these SNPs were included in the genomic breeding value estimation (GBLUP and ssGBLUP). To obtain correlations between traits, QTLs were assumed to also affect the other simulated traits, with contributions to other traits being calculated based on a Cholesky decomposition of the target correlation matrix (for details see MoBPS Guidelines, chapter 15; https://github.com/tpook92/MoBPS). For males, no phenotypes were generated but females were assigned phenotypes that were measured at different time points in life, depending on the trait. The laying performance was split into four time periods that were considered as four separate traits. Laying performance 1 and 2, egg weight, feed consumption, egg shell strength, hatchability, and mortality were available before the time of selection at 51 weeks. Although a mortality trait was considered, beyond that early mortality was not separately simulated. It was assumed that all females were kept until 72 weeks of life to measure further phenotypes, regardless of whether they were selected for breeding or not. Laying performance 3 and 4, a second measurement of egg weight, feed consumption, egg shell strength, hatchability, and mortality were recorded in this time frame and were, thus, only used for selection in the next cycle. For traits with multiple observations, the residual effects of phenotypes of the same trait at different time points were assumed to be independent of each other, not considering permanent environmental effects. In the scenarios with the shortened generation interval for females, it was assumed that no own phenotypes were available on the females selection candidates at the time of selection.

**Table 1:** Heritabilities, phenotypic standard deviations, correlations between traits, and economic weights of the simulated traits [9]

	ew	SD	1	2	3	4	5	6	7	8	9
Laying performance 1 (1)	3	22.7	*0.35*	0.16	− 0.05	− 0.10	− 0.20	− 0.14	− 0.06	0.05	0.05
Laying performance 2 (2)	6	4.8	0.06	*0.10*	0.73	0.58	− 0.30	0.05	− 0.05	0.15	− 0.05
Laying performance 3 (3)	6	7.0	− 0.01	0.47	*0.12*	0.85	− 0.20	0.09	− 0.08	0.15	− 0.05
Laying performance 4 (4)	9	7.3	− 0.07	0.27	0.32	*0.20*	− 0.20	0.10	− 0.12	0.20	− 0.08
Egg weight (5)	18	3.8	− 0.18	− 0.08	− 0.04	− 0.06	*0.75*	0.64	− 0.20	− 0.40	0.02
Feed consumption (6)	− 12	10.0	− 0.05	0.08	0.07	0.01	0.43	*0.50*	− 0.05	− 0.24	0.01
Egg shell strength (7)	7	7.0	0.05	0.13	0.11	0.10	− 0.12	− 0.04	*0.35*	0.08	0.02
Hatchability (8)	2	25.8	0.05	0.13	0.09	0.07	− 0.25	− 0.13	0.09	*0.26*	− 0.01
Mortality (9)	− 3	0.2	0.02	− 0.03	− 0.04	− 0.05	0.01	0.01	− 0.01	− 0.01	*0.03*

ew = relative economic weight per trait unit; SD = phenotypic standard deviation; heritabilities on diagonal; genetic correlations above diagonal; phenotypic correlations below diagonal

Selection was based on a multiple-trait index. Index weights on the EBV for the traits were calculated based on economic weights and correlations between traits [30] and scaled based on reliabilities [31, 32]. To save computing time, reliabilities were estimated based on the correlation between true and estimated breeding values (or phenotypes in case phenotypic selection was used) for each cohort. In all scenarios, mating of full and half siblings was not permitted.

### Breeding program

**Fig. 1:**
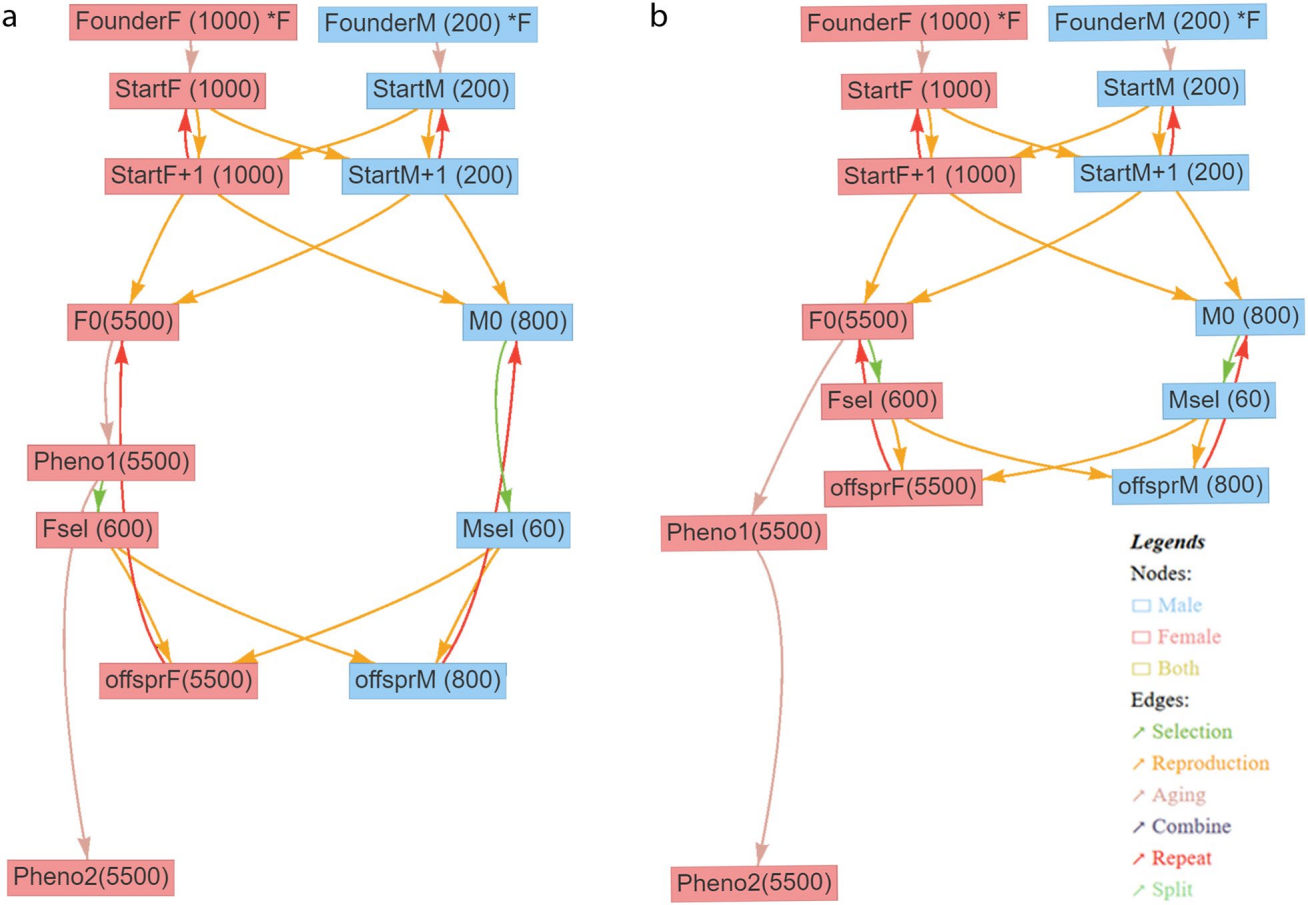
Simulated breeding program for generation intervals of 63 and 31.5 weeks. Schematic illustration of the simulated breeding program from the user interface MoBPSweb [28] for a generation interval of 63 weeks (**a**) and a generation interval of 31.5 weeks (**b**). Blue nodes represent cohorts of males and red nodes cohorts of females. Numbers of individuals are given in brackets. Edges represent breeding actions, with the color of the edge indicating the type of breeding action (see legend)

**Fig. 2:**
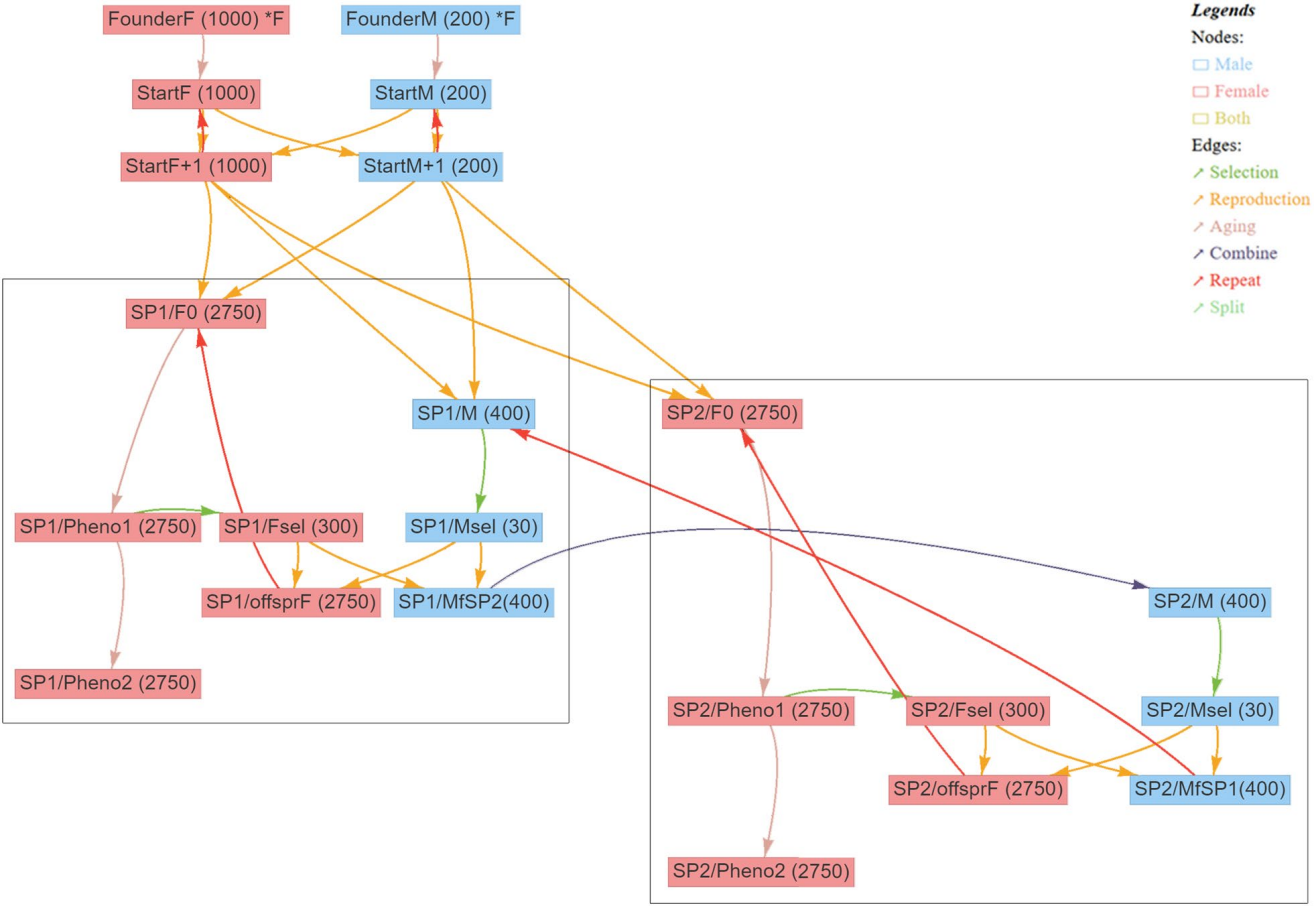
Simulated breeding program with two time-shifted subpopulations with different generation intervals for females and males. Schematic illustration of the simulated breeding program with two time-shifted subpopulations from the user interface MoBPSweb [28] for a generation interval of 63 weeks for females and a generation interval of 31.5 weeks for males. Blue nodes represent cohorts of males and red nodes cohorts of females. Numbers of individuals are given in brackets. Edges represent breeding actions, with the color of the edge indicating the type of breeding action (see legend)

A schematic overview of the basic breeding schemes in the different scenarios is given in Figs. 1 and 2. In the reference scenario, the generation interval was assumed to be 63 weeks [9]. In some scenarios, this was halved to 31.5 weeks, either for males and females or only for males. When the generation interval was reduced for both sexes, no major changes in the structure of the breeding program were needed, except that females were selected before own phenotypes became available. When reducing the generation interval only on the male side, the population was divided into two time-shifted populations (31.5 weeks apart) and young males were transferred to the other subpopulation where they were mated to the older females. Therefore, the phenotypes of females of a subpopulation can be used in their own selection, but they are not available when selecting male selection candidates from that subpopulation (Fig. 2).

In all modelled breeding programs, 60 males were selected from 800 selection candidates and 600 females were selected from 5500 selection candidates, except for one scenario which had a reduced number of female selection candidates. For scenarios with two time-shifted subpopulations, the two subpopulations were each half the size of the population in the base scenario. Compared to the simple reference breeding scheme with a generation interval of 63 weeks (Fig. 1a), the breeding scheme had to be adapted when shortening the generation interval to 31.5 weeks such the first own phenotypes for females became available after the time of selection (Fig. 1b). The JavaScript Object Notation (JSON) files from MoBPSweb (www.mobps.de) for all scenarios are given in Additional file 1 Input files S1 to S18.

### Simulated scenarios

In total eighteen different scenarios were simulated. An in-depth overview of selection methods and amount of genotyping per generation for all scenarios are given in Table 2. The first nine scenarios had the structure of the reference breeding program, with a generation interval of 63 weeks for both males and females (Fig. 1a). In the first scenario, the EBVs for the males were calculated using pedigree-based BLUP [33], while selection of females was based on own phenotypes (scenario 1). In the second scenario, it was assumed that pedigree-based BLUP breeding value estimation was also performed for female selection candidates (scenario 2). In scenario 3 to 6, the EBVs for the male selection candidates were based on ssGBLUP [5, 6], with different amounts of genotyping. In scenario 3, only the male selection candidates were genotyped. In scenarios 4 and 5 the 600 selected females were genotyped also, after their selection, while in scenario 6, 600 randomly chosen female selection candidates were genotyped before selection. In scenario 7, all male and female selection candidates were genotyped and, therefore, the male selection candidates were selected based on genomic BLUP (GBLUP) [3]. In these scenarios, except for scenario 5, females were nevertheless selected based on their phenotypes to distinguish in the simulations between the effect of the genotyped cohorts on the selection of males and the effect of the breeding value estimation for females. In scenario 5, selection was on ssGBLUP to investigate the effect of such selection for females without own genotypes. In scenario 8, selection of the females was based on GBLUP and all male and female selection candidates were genotyped. Scenario 9 was the same as scenario 8 but, to evaluate the potential of optimal contribution selection (OCS) [18] on the development of inbreeding in the population, OCS based on a pedigree-based relationship matrix was used by integrating the R-package optiSel in MoBPS [34] (method = “min.sKin” in optiSel). Besides this, the same assumptions were made as for scenario 8.

**Table 2:** Selection criteria and genotyped cohorts for the simulated scenarios

Scenario		Generation interval (weeks)		Selection method		Genotyped individuals (number genotyped in each generation)		OCS
		Male	Female	Male	Female	Male	Female	
Long generation interval								
1	longGI-m:Ped-f:Pheno	63	63	Pedigree	Phenotypic	–	–	No
2	longGI-m:Ped-f:Ped	63	63	Pedigree	Pedigree	–	–	No
3	longGI-m:ssGBLUP-f:Pheno-Geno:M	63	63	ssGBLUP	Phenotypic	Candidates (800)	-	No
4	longGI-m:ssGBLUP-f:Pheno-Geno:M&Fsel	63	63	ssGBLUP	Phenotypic	Candidates (800)	Selected (600)	No
5	longGI-m:ssGBLUP-f:ssGBLUP-Geno:M&Fsel	63	63	ssGBLUP	ssGBLUP	Candidates (800)	Selected (600)	No
6	longGI-m:ssGBLUP-f:Pheno-Geno:M&F600	63	63	ssGBLUP	Phenotypic	Candidates (800)	Random (600)	No
7	longGI-m:GBLUP-f:Pheno-Geno:M&F	63	63	GBLUP	Phenotypic	Candidates (800)	Candidates (5500)	No
8	longGI-m:GBLUP-f:GBLUP-Geno:M&F	63	63	GBLUP	GBLUP	Candidates (800)	Candidates (5500)	No
9	longGI-m:GBLUP-f:GBLUP-Geno:M&F-OCS	63	63	GBLUP	GBLUP	Candidates (800)	Candidates (5500)	Yes
Halved generation interval only for males (two time-shifted subpopulations)								
10	2SP-m:ssGBLUP-f:Pheno-Geno:M&Fsel	31.5	63	ssGBLUP	Phenotypic	Candidates (2 × 400)	Selected (2 × 300)	No
11	2SP-m:ssGBLUP-f:ssGBLUP-Geno:M&Fsel	31.5	63	ssGBLUP	ssGBLUP	Candidates (2 × 400)	Selected (2 × 300)	No
12	2SP-m:GBLUP-f:GBLUP-Geno:M&F	31.5	63	GBLUP	GBLUP	Candidates (2 × 400)	Candidates (2 × 2750)	No
13	2SP-m:GBLUP-f:GBLUP-Geno:M&F-OCS	31.5	63	GBLUP	GBLUP	Candidates (2 × 400)	Candidates (2 × 2750)	Yes
Short generation interval for both males and females								
14	shortGI-m:ssGBLUP-f:Ped-Geno:M	31.5	31.5	ssGBLUP	Pedigree	Candidates (800)	–-	No
15	shortGI-m:ssGBLUP-f:ssGBLUP-Geno:M	31.5	31.5	ssGBLUP	ssGBLUP	Candidates (800)	–-	No
16	shortGI-m:GBLUP-f:GBLUP-Geno:M&F	31.5	31.5	GBLUP	GBLUP	Candidates (800)	Candidates (5500)	No
17	shortGI-m:GBLUP-f:GBLUP-Geno:M&F-OCS	31.5	31.5	GBLUP	GBLUP	Candidates (800)	Candidates (5500)	Yes
18^1^	shortGI-m:GBLUP-f:GBLUP-Geno:M&F-n/2	31.5	31.5	GBLUP	GBLUP	Candidates (800)	Candidates (2750)	No

^1^Number of female selection candidates divided by two

Phenotypic = phenotypic selection; Pedigree = pedigree-based breeding value estimation; GBLUP = breeding value estimation based on genomic best linear unbiased prediction; ssGBLUP = breeding value estimation based on single-step GBLUP; OCS = use of Optimum Contribution Selection to minimize inbreeding

The acronym for a scenario is composed of the specification of the generation interval (longGI = long generation interval; 2SP = two time-shifted subpopulation with short generation interval only for males; shortGI = short generation interval), followed by the selection method for males (m) and females (f; Pheno = phenotypic selection; Ped = pedigree-based selection) and the specification which cohorts are genotyped (Geno; M = all male selection candidates, F = all female selection candidates, Fsel = selected females, F600 = 600 random female selection candidates). Further, OCS indicates that in the scenario OCS was used and n/2 indicates that number of female selection candidates was half compared to all other scenarios

Scenarios 10 through 13 used a generation interval of 63 weeks for females and a halved generation interval of 31.5 weeks for males by using the structure of two time-shifted subpopulations, as displayed in Fig. 2. The selection method in scenario 10 corresponds to that of scenario 4, with limited genotyping and phenotypic selection on the female side, while selection in scenario 12 corresponds to that of scenario 8 with genotyping of all male and female selection candidates, and scenario 11 corresponds to scenario 5 with limited genotyping and genomic selection on the female side. Finally, scenario 13 corresponds to scenario 9 with use OCS and genotyping of all animals and a halved generation interval for males.

In the last five scenarios (scenarios 14 to 18) the generation interval was shortened to 31.5 weeks for both males and females. Scenario 14 represents a low effort scenario for a shortened generation interval with the time of selection before own phenotypes are available by applying a pedigree-based BLUP for females and ssGBLUP for males, relying only on genotyping of male selection candidates. The scenario 15 is similar to scenario 14, but with ssGBLUP instead of pedigree-based BLUP for females. Additionally, in scenario 16, selection of males and females was based on GBLUP, with all male and female selection candidates genotyped, corresponding to scenarios 8 and 12 with the longer generation intervals. Additionally, in scenario 17, OCS was used in the breeding program with a halved generation intervals for both males and females, in concordance with scenarios 9 and 13. In scenarios 14 to 17 the numbers of individuals per generation were the same as in all other scenarios (Fig. 1b), resulting in a doubling of the total number of animals per unit of time, which increased housing and test capacity and costs of the breeding program. In scenario 18, the housing capacity on the female side was assumed to be equal to the long-generation scenarios and therefore the number of the females generated per cycle had to be halved as the previous cycle is only 31.5 weeks ahead and, therefore, still requires housing capacities.

For estimation of breeding values, the training population included animals of the last three breeding cycles. Heritabilities were assumed to be known to save computing time in retraining of prediction models for each new selection procedure. In the study of Wolc et al. [12], retraining was also compared with a scenario without retraining, in which the accuracy of the EBVs decreased in the subsequent breeding cycles. Therefore, the option to only use historic information as the basis for the EBVs was not further considered in the current study.

Except for scenario 18, the total number of animals included in breeding value estimation was kept constant, with the highest number of genotyped animals (18,900) in scenarios 7–8, 12–13, and 16–17 (Table 2).

### Evaluation of results

All scenarios were simulated for 630 weeks, which corresponds to 10 generations for the scenarios with the regular generation intervals and 20 generations for scenarios with the shortened generation interval. The results were reported as change from 63 weeks, which is the first simulated generation of scenarios with long generation intervals and the second generation of scenarios with shortened generation intervals, to the last simulated generation resulting in the same time span for each scenario. All results were given as an average of 20 independently performed simulations. Significances between scenarios were determined with a two-sample t-test [35].

In terms of genetic gain, results are presented based on their impact on a weighted index for each trait, as determined by the economic weights. Inbreeding levels [36] were calculated based on the share of the genome in identity-by-descent (IBD) [37]. Note that founder animals that were used to create the starting population were unrelated. The effects of different scenarios on both genetic gain and inbreeding were considered, with breeding programs that achieved the greatest genetic progress per unit of inbreeding considered as most sustainable. Results are also presented in two-dimensional box-plots, contrasting genetic progress and inbreeding rate for the considered scenarios. For each scenario, the median is indicated by a filled dot in the two-dimensional system of the coordinates for genetic gain (∆G; on the y-axis) and the increase in inbreeding (∆F; on the x-axis) for the breeding population from time point 63 weeks to 630 weeks. Additionally, the accuracy of EBVs was calculated based on the correlation between the true and estimated breeding values. The accuracy of phenotypic selection of hens was calculated by the correlation of the true breeding vales with the phenotypes. In both cases, the traits were weighted by their respective weights in the selection index. Accuracies were reported for the second to last simulated generation (generation 9) for the scenarios with long generation intervals (scenarios 1–9) and for the corresponding time point of the simulations for scenarios with shortened generation intervals (scenarios 10–18).

## Results

### Genotyping and breeding value estimation for females

**Fig. 3:**
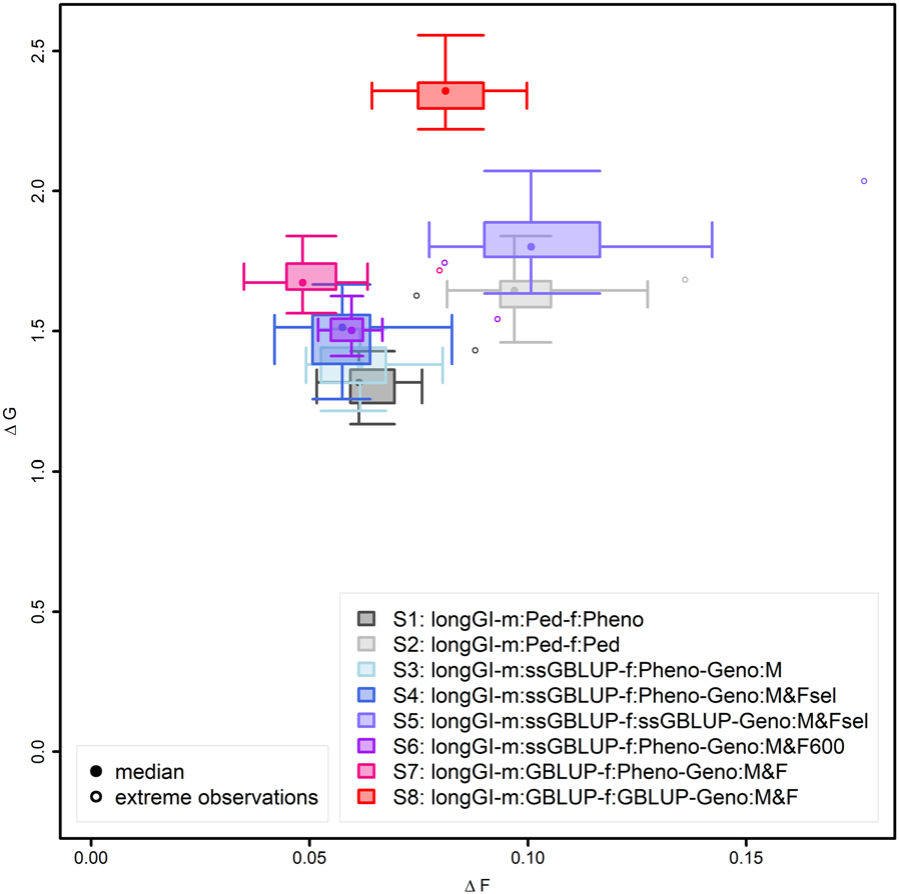
Change in genetic progress and inbreeding for different breeding value estimation methods and genotyping different cohorts. Ratio of genetic gain (∆G) and increase in inbreeding (∆F) for the breeding population from 63 to 630 weeks, displayed as a two-dimensional boxplot of 20 simulations. The borders of the box indicate the 25 and 75% quantiles for both axes (∆G and ∆F). The whiskers indicate either 2.5 standard deviations or, in case the maximum and minimum values are closer to the mean, these maximum or minimum values. Simulation replicates that were more than 2.5 standard deviations different from the mean are indicated as extreme values

In terms of genetic gain, in the scenarios with the regular generation interval, EBVs for females (scenarios 2, 5, and 8) showed to be highly advantageous compared to selection on own phenotype (scenarios 1, 3–4, and 6–7; Fig. 3). When using genomic selection only for selection of males (scenarios 3–4 and 6–7) or for selection of both males and females (scenarios 5 and 8), also genotyping females was found to be clearly advantageous (scenarios 4–9; Fig. 3).

Compared to phenotypic selection (scenario 1), selection on pedigree-based BLUP EBV for females (scenario 2) showed a very significant increase in genetic gain of + 23.9% (p ≤ 10^–10^; see Additional file 2 Table S1). Using ssGBLUP for selection of males and only genotyping male selection candidates (scenario 3) only led to a small improvement in genetic gain (4.1%; p = 0.09) compared to the baseline scenario 1. Compared to scenario 3, collecting additional genotypes on selected females (scenario 4), randomly chosen females (scenario 6), or all females (scenario 7) led to further very significant improvements of 7.3, 10.4, and 23.2%, respectively [for p-values see Additional file 2 Table S1]. Herein, the difference between the genotyping of the selected females (scenario 4) and the genotyping of randomly chosen selection candidates (scenario 6) was not significant (p = 0.182). Genetic gain increased very significantly by 24.8% (p ≤ 10^–10^) when also selecting females using ssGBLUP EBV and only genotyping selected females (scenario 4 vs. scenario 5) and by 39.4% (p ≤ 10^–10^), when genotyping all females and, consequently, selecting them based on GBLUP EBV (scenario 8 vs. scenario 7, Fig. 3).

The average accuracy of the breeding value estimation for the selection index based on pedigree-based BLUP was 0.55 for males (scenarios 1–2; Table 3) and 0.67 for females (scenario 2), compared to an accuracy of 0.64 for phenotypic selection of females. The accuracy of the EBV for males based on GBLUP increased from 0.65 when only genotyping male selection candidates (scenario 3) to 0.72 when also genotyping selected females (scenario 4). When the same number of randomly sampled females was genotyped instead, before the time of selection (scenario 6), the accuracy increased to 0.72. When genotyping all males and females (scenario 7), accuracy increased to 0.83 (Table 3).

**Table 3:** Accuracy of selection for male and female selection candidates with standard errors (SE) in brackets

Scenario	Selection method		Genotyped individuals (number genotyped of each generation)		Accuracy of selection^3^	
	Males	Females	Males	Females	Males	Females
Long generation interval						
1	Pedigree	Phenotypic	–	–	0.55 (0.021)	0.64 (0.009)*
2	Pedigree	Pedigree	–	–	0.55 (0.019)	0.67 (0.013)
3	ssGBLUP	Phenotypic	Candidates (800)	–	0.65 (0.019)	0.64 (0.007)*
4	ssGBLUP	Phenotypic	Candidates (800)	Selected (600)	0.72 (0.014)	0.64 (0.008)*
5	ssGBLUP	ssGBLUP	Candidates (800)	Selected (600)	0.76 (0.015)	0.67 (0.014)
6	ssGBLUP	Phenotypic	Candidates (800)	Random (600)	0.72 (0.011)	0.64 (0.006)*
7	GBLUP	Phenotypic	Candidates (800)	Candidates (5500)	0.83 (0.008)	0.64 (0.007)*
8	GBLUP	GBLUP	Candidates (800)	Candidates (5500)	0.83 (0.010)	0.84 (0.007)
9^2^	GBLUP	GBLUP	Candidates (800)	Candidates (5500)	0.84 (0.007)	0.84 (0.006)
Halved generation interval only for males (two time-shifted subpopulation)						
10	ssGBLUP	Phenotypic	Candidates (2 × 400)	Selected (2 × 300)	0.63 (0.021)	0.63 (0.009)*
11	ssGBLUP	ssGBLUP	Candidates (2 × 400)	Selected (2 × 300)	0.69 (0.022)	0.67 (0.015)
12	GBLUP	GBLUP	Candidates (2 × 400)	Candidates (2 × 2750)	0.79 (0.012)	0.84 (0.010)
13^2^	GBLUP	GBLUP	Candidates (2 × 400)	Candidates (2 × 2750)	0.80 (0.014)	0.84 (0.010)
Short generation interval for males and females						
14	ssGBLUP	Pedigree	Candidates (800)	–	0.53 (0.025)	0.31 (0.020)
15	ssGBLUP	ssGBLUP	Candidates (800)	–	0.53 (0.029)	0.37 (0.034)
16	GBLUP	GBLUP	Candidates (800)	Candidates (5500)	0.73 (0.013)	0.73 (0.010)
17^2^	GBLUP	GBLUP	Candidates (800)	Candidates (5500)	0.72 (0.014)	0.73 (0.011)
18^1^	GBLUP	GBLUP	Candidates (800)	Candidates (2750)	0.66 (0.019)	0.66 (0.016)

^*^Accuracy of phenotypic selection

^1^Number of female selection candidates divided by two

^2^OCS = use of Optimum Contribution Selection to minimize inbreeding

^3^SE for accuracies are calculated based on 20 independent replicates

In addition to genetic gain, the inbreeding per unit of time and the ratio between genetic gain and inbreeding were analyzed. In general, inbreeding rates were substantially higher when females were selected based on EBVs instead of phenotypes (scenario 2, scenario 5 and scenario 8; Fig. 3), with relative increases in inbreeding rates of 56.0% with no pedigree-based selection of females (scenario 1 vs. scenario 2), 82.5% with limited genotyping (scenario 4 vs. scenario 5), and 62.1% when genotyping all animals (scenario 7 vs scenario 8).

On the male side, the use of ssGBLUP (scenarios 3–4 and scenario–6–7) instead of pedigree BLUP (scenario 1) reduced inbreeding rates (Fig. 1). For example, genomic selection of males when only the male selection candidates were genotyped (scenario 3), resulted in a 4.6% lower inbreeding rate compared to selection on pedigree-based BLUP EBV (scenario 1). In addition to genotyping male candidates, only genotyping selected females their selection (scenario 4) resulted in a decrease of the inbreeding rates by 9.5%, whereas genotyping 600 randomly chosen females (scenario 6) resulted in an average decrease in inbreeding rates by 5.8% compared to scenario 1. When all female selection candidates were genotyped (scenario 7; Fig. 3) inbreeding rates were 22.2% lower than for scenario 1.

When considering the ratio between genetic gain and inbreeding (∆G/∆F), selection of females on pedigree-based BLUP EBV for males and females had the lowest—and thus worst—ratio of ∆G/∆F (scenario 2 vs. scenario 1: − 20.6%; Fig. 3). Genotyping of more individuals but selecting females based on own phenotypes led to a higher ratio of ∆G/∆F (scenario 3 vs. 1: + 9.1%; scenario 4 vs. 1: + 23.4; scenario 6 vs. 1: + 22.0%; scenario 7 vs. 1: + 64.8%) caused by increases in ∆G and simultaneous decreases in ∆F. Genotyping 600 random females led to a slightly lower ∆G/∆F than genotyping of the 600 selected females (scenario 6 vs. scenario 4). When selecting the females based on genomic EBV, the impact of genotyping a larger number of females was even more important to reach a higher ratio of ∆G/∆F than when selecting females based on own phenotypes (scenario 5 vs. 1: − 15.7%; scenario 8 vs. 1: 41.7%), since a larger number of genotyped females leads to both an increase in ∆G and a decrease in ∆F.

### Shortening generation intervals

**Fig. 4:**
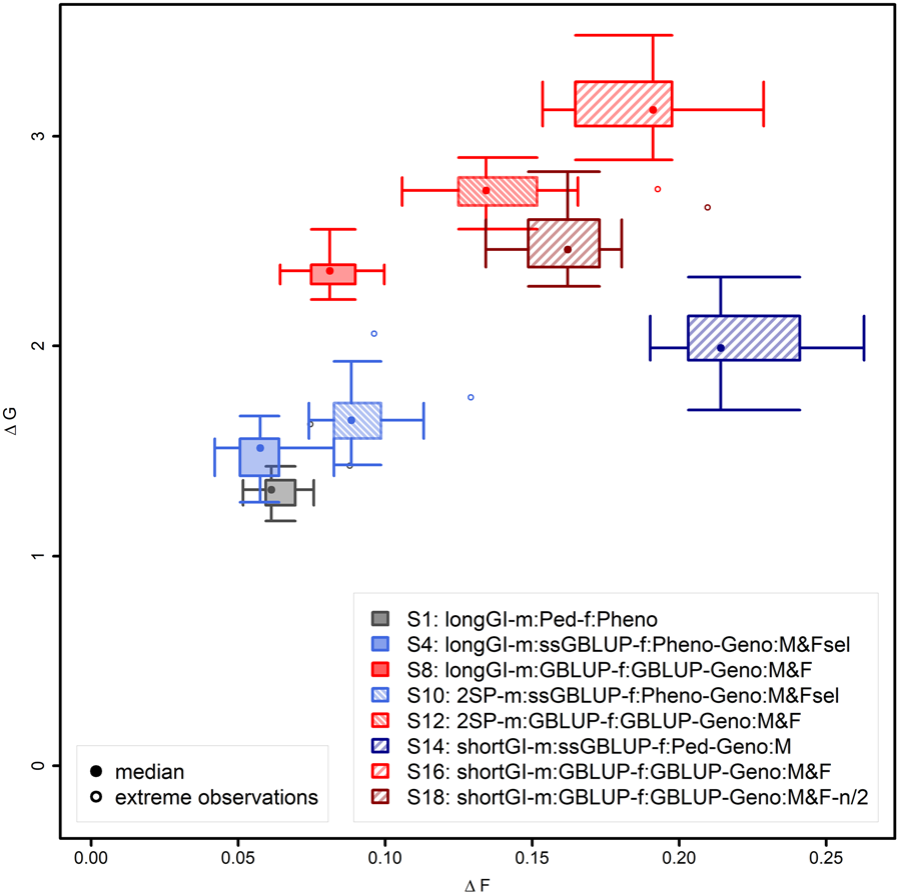
Change in genetic progress and inbreeding for breeding programs with shortened generation intervals. Ratio of genetic gain (∆G) and increase in inbreeding (∆F) for the breeding population from 63 to 630 weeks, displayed as a two-dimensional boxplot of 20 simulations. The median of each scenarios is indicated by a filled dot in the coordinates for ∆G to ∆F for the breeding population from 63 to 630 weeks. The borders of the box indicate the 25 and 75% quantiles for both axes (∆G and ∆F). The whiskers indicate either 2.5 standard deviations or, in case the maximum and minimum values are closer to the mean, these maximum or minimum values. Simulation replicates that were more than 2.5 standard deviations different from the mean are indicated as extreme values

Shortening the generation interval either only on the male side or for both males and females (scenario 10 and 12 and 14 and 16, respectively) was found to be advantageous in terms of genetic gain per unit of time as long as the number of selection candidates was not reduced (scenario 18; Fig. 4). When using a shortened generation interval for the male side of the breeding program only, very significant improvements were observed when using genomic selection also for females (scenario 8 vs. 12; + 16.0%; p ≤ 10^–10^), and also when selecting females based on own phenotype (scenario 4 vs. 10; + 13.6%; p = 5.2 × 10^–5^).

With a halved generation interval for both males and females (scenario 16 vs. 12/scenario 14 vs. 10) genetic gain increased by a further 15.5/21.3% (p ≤ 10^–10^/p = 3.4 × 10^–8^) when the number of selection candidates was constant (Fig. 4). However, when reducing the number of female selection candidates, (scenario 18), genetic gain was reduced by 8.5% (p = 4.2 × 10^–6^) compared to when only reducing the generation interval on the male side (scenario 12; Fig. 4), but genetic gain was increased by 6.1% (p = 0.001) compared to the corresponding scenario with generation intervals that were not shortened (scenario 8; Fig. 4).

Halving generation interval on the male side systematically reduced the accuracy of the EBVs (absolute difference between scenarios 4 and 10: 0.09; between scenarios 5 and 11: 0.07; between scenarios 8 and 12: 0.04; Table 3). In contrast, the accuracy of the EBVs for females was very similar for scenarios with different generation intervals (scenarios 5 and 11: 0.67; scenarios 8 and 12: 0.84; Table 3).

For all scenarios with a shortened generation interval, substantially higher increases of the inbreeding per year were observed compared to the corresponding breeding scheme with regular generation interval (scenario 4 vs. 10: 56.7%; scenario 8 vs. 12: 69.9%; scenario 10 vs. 14: 138.5%; scenario 12 vs. 16: 34.0%; Fig. 4). As the selection intensity in scenario 18, in which the number of female selection candidates was divided by two, was smaller than in scenario 16, relative increases in inbreeding per year were also smaller (scenarios 12 vs. 18: 16.7%; Fig. 4).

When considering the ratio ∆G/∆F, halving the generation interval only for males while selecting females based on own phenotype and genotyping male selection candidates as well as selected females, decrease ∆G/∆F by 27.5% (scenario 10 vs. 4; Fig. 4). This was mainly caused by an increase in ∆F. Also, due to the increase in ∆F in the scenarios with genomic selection of both males and females and genotyping all selection candidates, halving the generation interval for males led to a decrease of ∆G/∆F by 31.7% (scenario 12 vs. 8). When halving the generation interval for both males and females (scenario 16 vs. 12), ∆G/∆F decreased further by 13.8%. Reducing the number of female selection candidates by half had less influence on ∆G/∆F (scenario 18 vs. 16; − 9.1%), but is also accompanied by a reduction in ∆G.

**Fig. 5:**
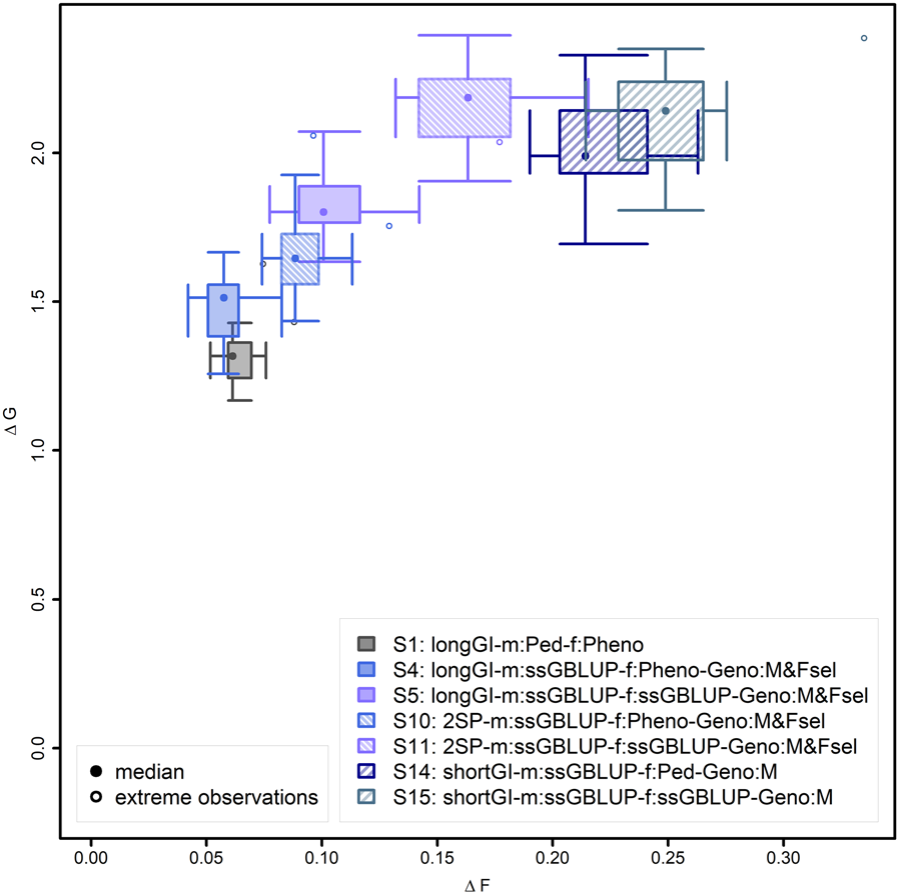
Change in genetic progress and inbreeding for pedigree-based and genomic breeding value estimation. Ratio of genetic gain (∆G) and increase in inbreeding (∆F) for the breeding population from 63 to 630 weeks, displayed as a two-dimensional boxplot of 20 simulations. The median of each scenarios is indicated by a filled dot in the coordinates for ∆G to ∆F for the breeding population from 63 to 630 weeks. The borders of the box indicate the 25 and 75% quantiles for both axes (∆G and ∆F). The whiskers indicate either 2.5 standard deviations or, in case the maximum and minimum values are closer to the mean, these maximum or minimum values. Simulation replicates that were more than 2.5 standard deviations different from the mean are indicated as extreme values

Genomic selection for females instead of phenotypic selection led to a very significant increase in genetic gain, both in the scenarios with long generation intervals and in the scenarios with a halved generation interval on the male side (scenario 5 vs. 4: 24.8%; scenario 11 vs. 10: 29.2%; both p ≤ 10^–10^). For the scenarios with halved generation interval for both males and females, genomic selection for females (scenario 15) could not be compared with phenotypic selection, since the phenotypes of the females were not available at the time of selection. However, compared to selection on pedigree-based BLUP EBV (scenario 14), the increase in genetic gain was 4.2% (p = 0.117; Fig. 5).

In addition to an increase in genetic gain, there was also an increase in inbreeding rates in these three considered comparisons (scenario 5 vs. 4, scenario 11 vs. 10, scenario 15 vs. 14). For the scenarios with long generation intervals, genomic selection of females increased the inbreeding rate by 82.5% (scenario 5 vs. 4) and for the scenario with halved generation intervals for males, the corresponding increase in inbreeding rate was 77.8% (scenario 11 vs. 10). In the scenarios with halved generation interval for both males and females, the use of genomic selection for females (scenario 15) led to a 14.2% higher inbreeding rate compared to using pedigree-based BLUP (scenario 14).

For ∆G/∆F, the scenarios with genomic selection of females (scenarios 5, 11, and 15) performed worse than the scenarios with phenotypic selection (scenarios 1, 4, and 10; Fig. 5). Comparing the same scenarios with selection of females on own phenotype versus ssGBLUP EBV, the difference in ∆G/∆F was 46.3% for the scenarios with long generation intervals (scenario 4 vs. 5) and 37.6% for the scenarios with halved generation interval for males (scenario 10 vs. 11). For the scenarios with halved generation intervals for males and females, ∆G/∆F was 9.6% lower for selection of females based on pedigree-based BLUP EBV instead of ssGBLUP EBV (scenario 14 vs. 15). For all considered generation intervals, an increase in ∆F caused a decrease in ∆G/∆F, even if ∆G also increased.

### Optimum contribution selection to reduce inbreeding

**Fig. 6:**
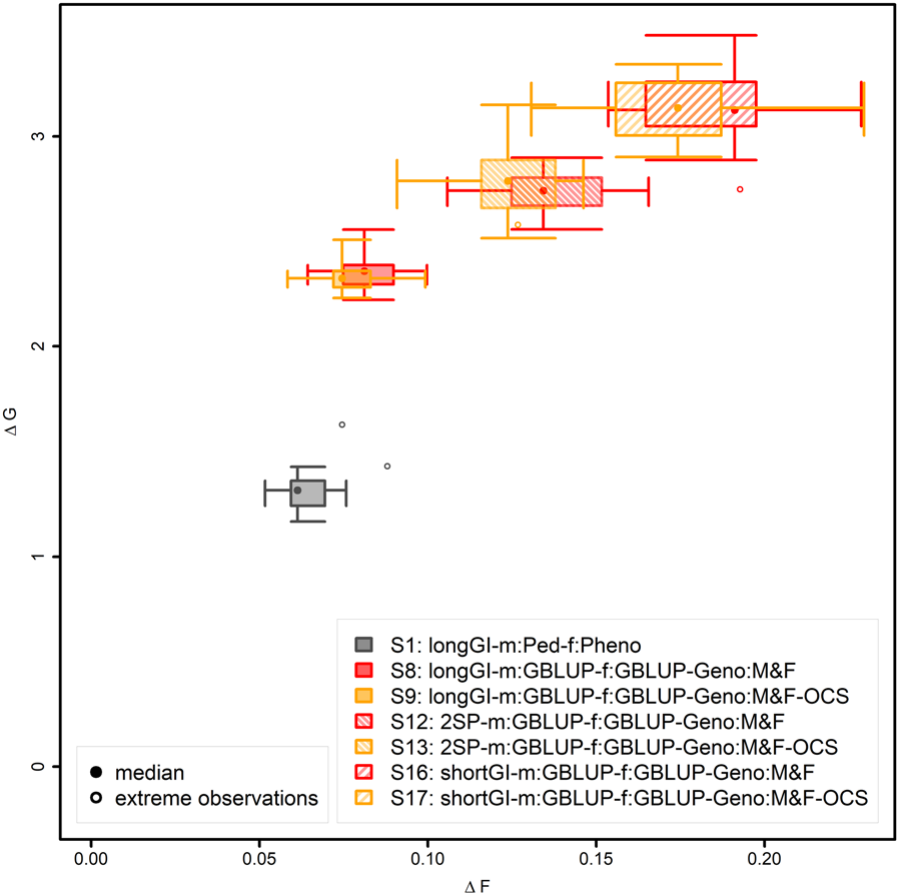
Change in genetic progress and inbreeding by the use of optimum contribution selection. Ratio of genetic gain (∆G) and increase in inbreeding (∆F) for the breeding population from 63 to 630 weeks displayed as a two-dimensional boxplot of 20 simulations. The median of each scenario is indicated by a filled dot in the coordinates for ∆G to ∆F for the breeding population from 63 to 630 weeks. The borders of the box indicate the 25 and 75% quantiles for both axes (∆G and ∆F). The whiskers indicate either 2.5 standard deviations or, in case the maximum and minimum values are closer to the mean, these maximum or minimum values. Simulation replicates that were more than 2.5 standard deviations different from the mean are indicated as extreme values

For all scenarios with OCS, no strong impact on genetic gain was observed compared to the corresponding breeding scheme without OCS (scenario 9 vs. 8: − 0.7%; scenario 13 vs. 12: 2.1%; scenario 17 vs. 16: − 1.0%; Fig. 6). The accuracy of EBV for females was not affected by the use of OCS. However, the use of OCS reduced inbreeding rates, in particular in the scenarios with shortened generation intervals (scenario 9 vs. 8: 3.1%; scenario 13 vs. 12: 10.0%; scenario 17 vs. 16: 7.3%; Fig. 6). Thus, the use of OCS moved ∆G/∆F in the desired direction by minimizing the rate of inbreeding while not negatively influencing genetic gain (scenario 9 vs. 8: 2.4%; scenario 13 vs. 12: 13.4%; scenario 17 vs. 16: 6.8%; Fig. 6). The highest effect of applying OCS in terms of inbreeding rates was observed in the scenarios with shortened generation interval only for males (scenario 13 vs. scenario 12).

## Discussion

### Simulations

In the current study, different designs of a layer breeding program that resulted from the shortening of generation intervals and different selection strategies were simulated and analyzed. In contrast to previous work using MoBPS [38–40], the designs used herein allowed for the explicit modelling of time-shifted subpopulations to allow for shortening the generation interval only for males. The different breeding strategies considered allowed for the quantitative assessment of the effect of complex breeding decisions on genetic progress, inbreeding, and accuracy of EBV. The ratio ∆G/∆F, which is a major determinant of the sustainability of a closed breeding program, was used to compare the different scenarios. This approach is much more flexible with regard to balancing short-term and long-term selection responses than the comparison of genetic gain under a fixed rate of inbreeding per year, as considered e.g. by Quinton et al. [41] or Wolc et al. [12], and thus allows for a more in-depth analysis of layer breeding programs.

Phenotypes for hens were simulated for egg weight, feed consumption, egg shell strength, hatchability, and mortality at 51 and 72 weeks of age. It was assumed that the residual effects of phenotypes of the same trait at different time points were independent of each other. Any bias in accuracy of EBV that results from this assumption only affects ancestors of selection candidates and not selection candidates themselves, thus its effect on results is expected to be minor, and all scenarios should be similarly affected by this.

### Genotyping and breeding value estimation for females

The simulations show that, in terms of genetic gain, all considered scenarios that used genomic selection outperformed the reference scenario with pedigree-based BLUP EBV for the selection of males and phenotypic selection of females. This result is consistent with results based on deterministic calculations of accuracy of EBV in laying hens [9, 12]. However, our study additionally allows to quantify relative changes between scenarios in terms of genetic gains, inbreeding, and accuracies of EBV in complex breeding schemes with overlapping generations.

Note that pedigree-based selection did not perform well, particularly in terms of inbreeding, which can partly be attributed to pedigree-based selection having little or no power to select within families [42, 43]. In terms of inbreeding, this problem can be overcome by limiting the number of animals selected per family. Although it would have been possible to select females with a more sophisticated approach than phenotypic selection in some scenarios, scenarios with phenotypic selection of females were important to isolate the effect of the switch from a pedigree-based to genomic breeding value estimation on the male side. While the use of pedigree-based BLUP led to slightly higher genetic gains compared to phenotypic selection, for the use of pedigree-based BLUP a substantial increase in inbreeding rates and thus a much worse ratio ∆G/∆F was observed compared to phenotypic selection. Genomic selection led to higher genetic gains and a much smaller increase in inbreeding rates than pedigree-based BLUP, which has been reported before [44], especially in the context of layer chickens [12].

Jiménez-Montero et al. [45] observed that genotyping randomly chosen animals rather than genotyping the individuals with the best phenotypes or EBV resulted in higher accuracies of EBV. In contrast, in the current study, no significant differences in genetic gains or accuracies of EBVs were observed between genotyping the selected females or randomly genotyping the same number of females, indicating that if differences exist, they are small compared to the variance in accuracies between replicates. Our simulations do, however, indicate that the variance of genetic gain appeared to be smaller when genotyping random rather selected animals, which could be an additional criterion when deciding with strategy to use in practise. These observations should, however, be considered with caution as only 20 replicates were performed and differences were not statistically significant (based on an F-test [46]).

Genotyping all female selection candidates was found to have the greatest effect on genetic gain. Thus, when 6300 individuals were genotyped per generation its potential benefits must be weighed against the additional costs. We only considered three very basic levels of genotyping (only males, males and selected females, all), but results from these scenarios can be used to approximate outcomes of different proportions of individuals genotyped. For example, genotyping just 10.9% of females additionally to all male selection candidates increased the accuracy of EBV from 0.65 to 0.72, while genotyping the remaining 89.1% of females led to a relatively smaller additional increase in accuracy, to 0.83 (Table 3), indicating reduced marginal benefits when some of the females are already genotyped. As greater accuracy directly translates into genetic gain, increasing genotyping in practical breeding programs is worth further investigation, in particular as genotyping costs are decreasing and genotyping is beneficial both in terms of reduced inbreeding rates and higher genetic gains. Thus, a more in-depth analysis for economic optimization of the breeding program is warranted.

As expected, the accuracy of EBV increased with the number of genotyped animals [47]. There were substantial differences between selection on pedigree-based and genomic BLUP EBV when non-phenotyped animals were selected, resulting in lower genetic gain when using pedigree-based evaluation, which is in line with previous studies [11, 48]. At the same time, genomic selection of males resulted in lower inbreeding rates, which highlights the potential of genomic BLUP EBV to identify within family differences, which is a valuable asset for sustainable breeding.

### Shortening the generation interval

Wolc et al. [12] suggested to use young genomic selected males and older females as breeding animals. To our knowledge a breeding scheme with two time-shifted subpopulations and a halved generation interval only for males has not been considered further in the literature. Wolc et al. [12] criticised the use of young females in a setting of a commercial breeding program as not very practical due to the later sexual maturity of some hens and potential problems with egg size.

Disregarding these potential practical problems, halving the generation interval of either only males or both males and females was found to have the potential to increase the genetic gain in our study, even if a part of this advantage was counteracted by lower accuracies of the EBVs, which was particularly problematic when using pedigree-based EBVs. In contrast to Sitzenstock et al. [9], who assumed that performance testing cost per generation was kept constant when shortening the generation interval of the females, the current study also considered housing capacity to be restricted. In this scenario, the number of female selection candidates was halved, which had a large negative impact on genetic gain. Overall, reducing the generation interval for both sexes was only favourable when females were also genotyped and housing and phenotyping capacities were increased. Otherwise, the breeding program with two time-shifted populations was favourable, both in terms of higher genetic gain and lower inbreeding per year. A reason for the larger benefit of halving the generation interval on the male side versus also for females could be that males cannot be phenotyped and hence the accuracy of their EBV is not affected as much as for the female side.

When analyzing different design options of a breeding program, it is important to consider not only genetic gain but also inbreeding rate per year, as its negative effects such as inbreeding depression and loss of genetic diversity need to be avoided [49]. According to Leroy [49] production traits in livestock are affected more by inbreeding depression than e.g. conformation traits. In pure lines, both reproductive and productive traits can be negatively influenced by inbreeding. Sewalem [15] reported negative effects of inbreeding on egg number and sexual maturity in several White Leghorn layer lines. In that study, a negative effect of inbreeding on reproductive traits was only reported for one of the three investigated layer line. Additionally, inbreeding as an indicator for the remaining genetic diversity is also an indicator for potential long-term selection gain. We simulated breeding programs only for a restricted time period of 630 weeks and purely additive quantitative traits. Thus, genetic gains were basically constant over time, which may not occur though in a long term study. The effect of a higher increase of the inbreeding per year due to shortening the generation interval is already known from, e.g., cattle breeding [50]. In the current study, especially halving the generation interval for both males and females as well as the shortening of the generation interval only for males while selecting the females phenotypically has been shown to be disadvantageous in terms of the ratio ∆G/∆F. When considering to implement one of these scenarios, additional options to limit the increase in inbreeding should be considered, such as increasing selection proportions or using OCS [18].

For all simulated generation intervals, genomic selection for females not only led to a greater increase in genetic gain, but also to higher inbreeding, compared to scenario with the same design but selection based on phenotypes or pedigree BLUP EBVs, respectively.

### Optimum contribution selection to reduce inbreeding

In the present simulations, the use of OCS [18] resulted in slightly lower inbreeding rates, while no mentionable negative effect on genetic gains was observed, confirming results from previous studies such as König et al. [17]. In the current study, only the use of OCS to minimize inbreeding from the set of selection candidates was considered. The use of the OCS framework to obtain maximum genetic gain under a restricted inbreeding rate would also be possible but was not considered here.

In all scenarios for which OCS was considered, the use of OCS led to an improved ∆G/∆F ratio, thus, indicating the sustainability of the approach. Substantial improvements were obtained for scenarios with short generation intervals and therefore higher inbreeding rates. However, the use of OCS was not able to fully offset the effect of shortening the generation interval on the increase in inbreeding per year.

Henryon et al. [51] showed that the benefits of OCS were in general relatively robust to restrictions such as pre-selection of sires or only being applied for the selection of males. This suggests that the results in regard to OCS of this simulation study should also be valid for even more sophisticated selection strategies.

OCS was based on genomic EBVs, which was shown by Clark et al. [52] to reach higher genetic gains than pedigree-based EBVs when restricting inbreeding. However, the pedigree relationship matrix was used for OCS, which, according to Clark et al. [52], should not affect the results negatively as long as the population structure does not include large full sib families, which is not the case in the laying hen breeding programs considered here.

## Conclusions

Compared to phenotypic selection, selection of hens on EBV (pedigree, GBLUP or ssGBLUP) was found to be highly advantageous in terms of genetic gain, but in particular selection on pedigree-based EBVs also led to considerably higher inbreeding rates. Genotyping of hens in combination with genomic selection was found to be advantageous, as genetic gains were further increased and inbreeding rates were much lower than for selection on pedigree-based EBV. Shortening the generation interval only for males by the use of time-shifted subpopulations was shown to be beneficial when at least some females were genotyped and was even superior to using a short generation interval for both sexes when housing capacities were kept at the same level. Thus, shortening the generation interval for females is only beneficial when a sufficient proportion of hens is genotyped and housing capacities are increased.

Using optimum contribution selection to minimize inbreeding yielded no significant changes in genetic gain, whereas the inbreeding rates could be reduced in the scenarios with shortened generation intervals. However, the reduction in inbreeding was not sufficient to fully compensate for the increase in inbreeding per year when halving generation intervals. Nevertheless, the use of OCS is highly recommended because it comes at virtually no cost.

Finally, the ability to model breeding programs to quantitatively assess the consequences of various design questions, such as the model used for breeding value estimation, the proportion of genotyped animals and the length of the generation interval, was showcased in the current study through stochastic simulations. Because simulation is not costly or time consuming, yet allows for broad and extensive conclusions, this should be an important consideration before practical implementation of a breeding program.

## Supplementary Information

Additional file 1: Input files S1 to S18. Input files for the simulation of scenario 1 to enter in MoBPSweb (www.mobps.de).

Additional file 2: Table S1. P-values of significance test (two sample t-test) between true breeding values of all scenarios.

## Data Availability

All data generated or analysed during this study are included in this published article and its supplementary information files.
