# Supplementary material for: Analysis of different genotyping and selection strategies in laying hen breeding programs
Source: Genet Sel Evol. 2025 Apr 7;57:18. doi: 10.1186/s12711-025-00948-4 (PMC11974122; doi:10.1186/s12711-025-00948-4)
Supplement: Supplementary file 2 — Additional file 2: Table S1. P-values of significance test (two sample t-test) between true breeding values of all scenarios. [file 12711_2025_948_MOESM2_ESM.pdf]

Table S1 P-values of significance test (two-sample t-test) between true breeding values of all scenarios.

Scenario	S1	S2	S3	S4	S5	S6	S7	S8	S9	S10	S11	S12	S13	S14	S15	S16	S17	S18
**S1:** 63w_mPed_fPheno		$< 10^{-10}$	0.08635	0.00011	$< 10^{-10}$	$1.0 * 10^{-7}$	$< 10^{-10}$	$< 10^{-10}$	$< 10^{-10}$	$8.4 * 10^{-10}$	$< 10^{-10}$	$< 10^{-10}$	$< 10^{-10}$	$< 10^{-10}$	$< 10^{-10}$	$< 10^{-10}$	$< 10^{-10}$	$< 10^{-10}$
**S2:** 63w_mPed_fPed			$< 10^{-10}$	$1.7 * 10^{-5}$	$7.4 * 10^{-7}$	$4.1 * 10^{-5}$	0.03079	$< 10^{-10}$	$< 10^{-10}$	0.34205	$< 10^{-10}$	$< 10^{-10}$	$< 10^{-10}$	$5.7 * 10^{-10}$	$< 10^{-10}$	$< 10^{-10}$	$< 10^{-10}$	$< 10^{-10}$
**S3:** 63w_mGBLUP_fPheno_GenoM				0.00427	$< 10^{-10}$	$2.6 * 10^{-6}$	$< 10^{-10}$	$< 10^{-10}$	$< 10^{-10}$	$2.0 * 10^{-8}$	$< 10^{-10}$	$< 10^{-10}$	$< 10^{-10}$	$< 10^{-10}$	$< 10^{-10}$	$< 10^{-10}$	$< 10^{-10}$	$< 10^{-10}$
**S4:** 63w_mGBLUP_fPheno_GenoM&Fsel					$< 10^{-10}$	0.18215	$6.1 * 10^{-8}$	$< 10^{-10}$	$< 10^{-10}$	$5.2 * 10^{-5}$	$< 10^{-10}$	$< 10^{-10}$	$< 10^{-10}$	$< 10^{-10}$	$< 10^{-10}$	$< 10^{-10}$	$< 10^{-10}$	$< 10^{-10}$
**S5:** 63w_mGBLUP_fGBLUP_GenoM&Fsel						$< 10^{-10}$	$8.1 * 10^{-5}$	$< 10^{-10}$	$< 10^{-10}$	0.00068	$1.4 * 10^{-9}$	$< 10^{-10}$	$< 10^{-10}$	0.00025	$8.3 * 10^{-7}$	$< 10^{-10}$	$< 10^{-10}$	$< 10^{-10}$
**S6:** 63w_mGBLUP_fPheno_GenoM&F600							$1.1 * 10^{-8}$	$< 10^{-10}$	$< 10^{-10}$	0.00036	$< 10^{-10}$	$< 10^{-10}$	$< 10^{-10}$	$< 10^{-10}$	$< 10^{-10}$	$< 10^{-10}$	$< 10^{-10}$	$< 10^{-10}$
**S7:** 63w_mGBLUP_fPheno_GenoM&F								$< 10^{-10}$	$< 10^{-10}$	0.63551	$< 10^{-10}$	$< 10^{-10}$	$< 10^{-10}$	$1.4 * 10^{-8}$	$< 10^{-10}$	$< 10^{-10}$	$< 10^{-10}$	$< 10^{-10}$
**S8:** 63w_mGBLUP_fGBLUP_GenoM&F									0.53025	$< 10^{-10}$	$4.6 * 10^{-6}$	$< 10^{-10}$	$3.3 * 10^{-10}$	$2.4 * 10^{-8}$	$3.5 * 10^{-6}$	$< 10^{-10}$	$< 10^{-10}$	0.00114
**S9:** 63w_mGBLUP_fGBLUP_GenoM&F_OCS										$< 10^{-10}$	$2.3 * 10^{-5}$	$< 10^{-10}$	$1.3 * 10^{-10}$	$6.9 * 10^{-8}$	$1.2 * 10^{-5}$	$< 10^{-10}$	$< 10^{-10}$	0.00040
**S10:** 2SP_mGBLUP_fPheno_GenoM&Fsel											$< 10^{-10}$	$< 10^{-10}$	$< 10^{-10}$	$3.4 * 10^{-8}$	$1.6 * 10^{-10}$	$< 10^{-10}$	$< 10^{-10}$	$< 10^{-10}$
**S11:** 2SP_mGBLUP_fGBLUP_GenoM&Fsel												$< 10^{-10}$	$< 10^{-10}$	0.0101	0.34310	$< 10^{-10}$	$< 10^{-10}$	$1.1 * 10^{-8}$
**S12:** 2SP_mGBLUP_fGBLUP_GenoM&F													0.22600	$< 10^{-10}$	$< 10^{-10}$	$< 10^{-10}$	$< 10^{-10}$	$4.2 * 10^{-6}$
**S13:** 2SP_mGBLUP_fGBLUP_GenoM&F_OCS														$< 10^{-10}$	$< 10^{-10}$	$6.7 * 10^{-8}$	$1.8 * 10^{-7}$	$4.3 * 10^{-6}$
**S14:** 31.5_mGBLUP_fPed_GenoM															0.11649	$< 10^{-10}$	$< 10^{-10}$	$< 10^{-10}$
**S15:** 31.5_mGBLUP_fGBLUP_GenoM																$< 10^{-10}$	$< 10^{-10}$	$5.4 * 10^{-9}$
**S16:** 31.5_mGBLUP_fGBLUP_GenoM&F																	0.51998	$< 10^{-10}$
**S17:** 31.5_mGBLUP_fGBLUP_GenoM&F_OCS																		$< 10^{-10}$
**S18:** 31.5_mGBLUP_fGBLUP_GenoM&F_n/2																		
